# Diagnostic value of the combined use of radial probe endobronchial ultrasound and transbronchial biopsy in lung cancer

**DOI:** 10.1111/1759-7714.13425

**Published:** 2020-04-16

**Authors:** Sang Chul Lee, Eun Young Kim, Joon Chang, Sang Hoon Lee, Chang Hoon Han*

**Affiliations:** ^1^ Division of Pulmonology, Department of Internal Medicine National Health Insurance Corporation Ilsan Hospital Goyang South Korea; ^2^ Division of Pulmonology, Department of Internal Medicine Yonsei University College of Medicine Seoul South Korea

**Keywords:** Biopsy, bronchoscopy, fluoroscopy, lung cancer

## Abstract

**Background:**

Although the use of radial endobronchial ultrasound (R‐EBUS) with a guide sheath has shown improved diagnostic capability in peripheral pulmonary lesions, its utility is still low due to variable performance. To overcome its limitation, we evaluated the feasibility and efficacy of R‐EBUS combined with transbronchial biopsy (TBB) under fluoroscopic guidance.

**Methods:**

We retrospectively reviewed medical records of 74 patients with non‐small cell lung cancer (NSCLC) who underwent R‐EBUS combined with TBB or TBB alone as a diagnostic technique. Subjects were grouped according to the diagnostic modality used (R‐EBUS combined with TBB vs. TBB alone). Each group was matched for age, sex, and location of the biopsy. The chi‐square test and paired *t*‐test were used to compare characteristics and identify factors that affected the diagnostic yield.

**Results:**

The mean age of the study cohort was 67.4 ± 12.8 years, with 21 (56.8%) men and 16 (43.2%) women in each group. The lesion size was significantly smaller in the R‐EBUS group (23.6 vs. 33.9, *P* < 0.001). The diagnostic yield with the combined use of R‐EBUS and TBB (27/37, 72.9%) was significantly higher than that with standard TBB alone (22/37, 59.4%). Lung lesions with a positive bronchus sign were associated with a higher diagnostic yield (odds ratio = 3.52 [1.17–10.62]; *P* = 0.025).

**Conclusions:**

The combination of R‐EBUS with TBB resulted in a higher diagnostic yield than either technique alone. Thus, the addition of R‐EBUS biopsy would be helpful to improve the diagnostic yield of TBB.

**Key points:**

**Significant findings of the study:**

The combination of R‐EBUS with TBB under fluoroscopic guidance improved the diagnostic yield of PPLs compared to TBB alone. A tissue diagnosis was more likely in pulmonary lesions with the air‐bronchus sign.

**What this study adds:**

The use of R‐EBUS could help improve the low diagnostic yield of TBB under fluoroscopic guidance without increasing the incidence of complications.

## Introduction

Lung cancer represents a major worldwide disease burden.^1^ According to Global Cancer Statistics 2018, lung cancer is a leading cause of newly diagnosed cancer and deaths across 20 regions of the world.^2^ Despite the introduction of preventive strategies such as tobacco control, new diagnostic modalities, and therapeutic agents, the incidence of cancer and cancer‐related mortality rates are expected to increase.^3^

Recently, with the introduction of low‐dose helical computed tomography (CT) for lung cancer screening, the detection rate of peripheral pulmonary lesions (PPLs) has increased.^4^ Thus, there has been an increasing need to acquire histopathological specimens from PPLs. In addition to improving the survival rate through the early diagnosis of lung cancer, histopathology of PPLs also facilitates decision‐making regarding the use of new therapeutic agents such as immunotherapy.^5^

Conventionally, transbronchial biopsy (TBB) under fluoroscopic guidance has been widely used in the diagnosis of PPLs since the 1970s.^6^ Performing biopsies under real‐time imaging of the lesions is a major advantage of TBB. However, determining the three‐dimensional location of the lesion using only a two‐dimensional fluoroscopic image remains a challenging task. In particular, when the target area of the biopsy overlaps with other pulmonary structures, it is difficult to determine the precise location. Moreover, the diagnostic yield of TBB varies from 14% to 75%, which is lower than that of transthoracic needle aspiration.^7^, ^8^

Approximately 20 years ago, advanced bronchoscopic modalities, such as endoscopic navigation bronchoscopy (ENB), thin bronchoscopy, and radial endoscopic ultrasound (R‐EBUS), were introduced to overcome the shortcomings of these conventional techniques.^9^, ^10^ The 2013 American College of Chest Physicians guidelines recommend radial EBUS as an alternative to conventional bronchoscopy if the equipment and expertise are available.^11^

R‐EBUS using thin bronchoscopy has a higher diagnostic yield (51%–92%) and lower complication rate than conventional bronchoscopy.^12^, ^13^ Under ultrasound guidance, the operator can perform a biopsy while observing the PPL in real‐time. However, the equipment required for this procedure is expensive. Furthermore, the ability to accurately target the lesion may be highly dependent on the skills and knowledge of bronchial anatomy of the operator.^14^, ^15^, ^16^

Therefore, the purpose of this study was to determine the improved diagnostic capability of R‐EBUS when combined with conventional TBB in the diagnosis of PPLs.

## Methods

### Study design and patient selection

This study was conducted at the Severance Hospital, Seoul, Korea. A retrospective review of electronic medical records was conducted on consecutive lung cancer patients at the pulmonary division of the hospital from August 2017 to November 2018. During the study period, TBB was performed in 432 lung cancer patients. Among this cohort, 37 patients underwent TBB combined with R‐EBUS biopsy. They were matched with the remaining 395 patients who underwent TBB under fluoroscopic guidance alone according to age, sex, smoking status, and characteristics of the lesion, including location, type, and size. Among the 395 patients, we selected cases highly suspected of lung cancer on chest CT to reduce the possibility of including those with benign diseases. We also excluded cases with signs of infection such as fever, hypotension, leukocytosis, or elevated C‐reactive protein levels. Even if the study subjects' bronchoscopic biopsy revealed a benign pathology, they were followed up for six months. The diagnosis of lung cancer was made by using follow‐up images, multidisciplinary consultation, as well as another modality of biopsy such as CT‐guided needle aspiration biopsy or surgical biopsy.

### Ethics approval

This study was approved by the institutional review board of the Severance Hospital (Approval number 4–2019‐1106).

### Bronchoscopic procedures

**Figure 1:**
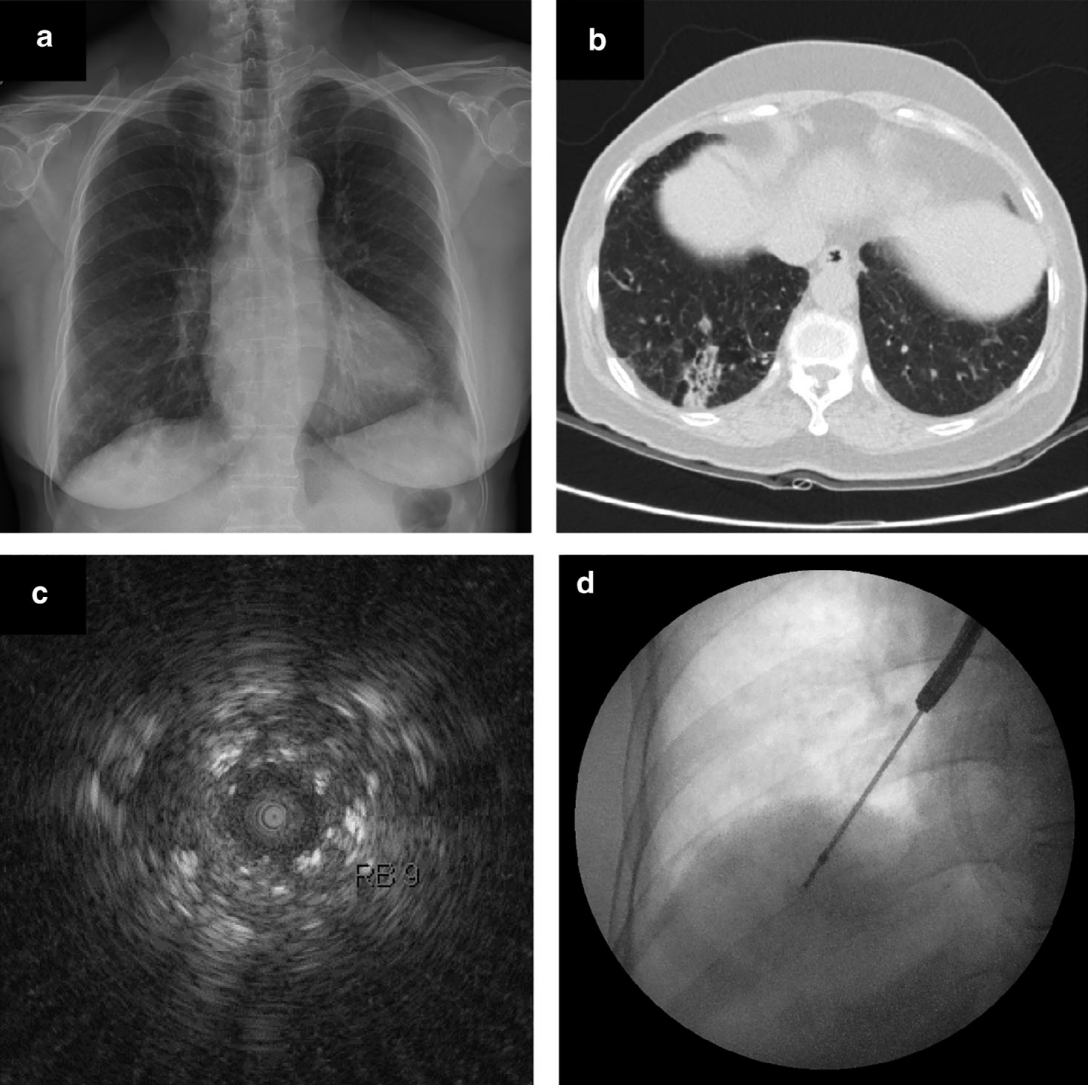
Case of a 74‐year‐old man with a consolidation in the right lower lobe.(**a**) Chest X‐ray showing consolidation at the right lower lobe. (**b**) Computed tomography (CT) scan showing a 38 mm peripheral lung mass. (**c**) Radial probe endobronchial ultrasound (R‐EBUS) in the lateral basilar segment of the right lower lobe revealing a mixed blizzard sign containing a heterogeneous acoustic shadow with hyperechoic dots, linear arcs, and vessels. (**d**) Fluoroscopic image during transbronchial biopsy through a guide sheath.

Bronchoscopy was performed by six fellowship trainees of the pulmonology division of our institution. Each fellowship trainee underwent a training period of 4–15 months and had performed a minimum of 100 TBBs. Both TBB and R‐EBUS were performed under the supervision of an expert faculty member with previous experience with the procedure. A thin bronchoscope (EB‐P290; Olympus) with a 4.2 mm outer diameter and a 2.0 mm working channel was used in both groups. EBUS was performed using an endoscopic ultrasound system (UM‐S20‐17S; Olympus, Japan), equipped with a 20‐MHz mechanical radial‐type probe. Prior to sedation, topical anesthesia of the larynx was applied for 5 minutes using a 2% lidocaine spray. To perform bronchoscopy under deep sedation, intravenous midazolam (2–5 mg depending on patient's bodyweight) and fentanyl (50 mcg) were administered. After insertion of the bronchoscope through the mouth, the trachea and whole bronchi were first inspected. The bronchoscope was placed on the segmental bronchus close to the PPL. A radial probe was then inserted through the working channel to advance into the segmental bronchus near the PPL to localize the lesion. This process was completed under fluoroscopic guidance to facilitate visualization. After localization using EBUS, the probe was removed with the guide sheath remaining on the peripheral lesion. Subsequently, biopsy forceps (Olympus, 1.5 mm portion diameter) were introduced via the guide sheath to perform pathological examination. After performing four biopsies using R‐EBUS, both the radial probe and guide sheath were removed via the working channel. The target site was located using typical radial EBUS images that have previously been described for solid nodules, and the blizzard sign for nodules demonstrating ground‐glass opacity (GGO).^17^ A representative case of R‐EBUS is shown in Fig 1. Afterwards, four biopsies were done only after confirming the approximate placement of the TBB forceps (Olympus, 1.9 mm portion diameter) to the PPL under fluoroscopy. With regard to the TBB group, only TBB was completed eight times under fluoroscopic guidance without prior R‐EBUS biopsy.

### Complications

Patients were hospitalized for more than two days for the bronchoscopy procedure and monitored for complications during the hospital stay. They were specifically monitored for the development of hemoptysis, pneumothorax, fever, desaturation, chest pain, and respiratory failure. In‐hospital mortality among the study subjects was also recorded. Chest radiography was performed within three hours after the procedure to check for possible pneumothorax and TBB‐related bleeding. Desaturation was defined as a drop in oxygen saturation to <95% during or after the procedure.

### Statistical analysis

Data analysis was completed on demographic and clinicopathologic variables. Descriptive statistics are reported as the mean ± standard deviation, and categorical variables as frequencies and proportions. Differences between the standard TBB and R‐EBUS with TBB groups were analyzed using the Student's *t*‐test, chi‐square test, or Fisher's exact test. We performed univariate and multivariate logistic regression analyses to assess the significance of lesion characteristics as an independent predictive factor for diagnostic yield. Statistical analyses were performed using the IBM SPSS software, version 25.0 (IBM Co., Armonk, NY, USA).

## Results

### Characteristics of study subjects and procedures

The study cohort consisted of 37 patients in each group, including 21 (56.8%) men and 16 (43.2%) women. The mean age of the study cohort was 67.4 ± 12.8 years. Comorbidities such as hypertension, diabetes, and tumor marker (carcinoembryonic antigen and Cyfra 21–1) levels were not significantly different between the two groups. The mean duration of the bronchoscopy procedure was 42.9 ± 16.4 minutes in all patients. The mean procedural time was longer in the R‐EBUS‐TBB group than in the TBB group (47.6 ± 15.3 vs. 38.1 ± 16.2 minutes); however, the difference was not statistically different (*P* = 0.743) (Table 1).

**Table 1:** Patient and lesion characteristics

Variable	All subjects	Standard TBB	R‐EBUS‐TBB	*P*‐value
Male sex	42 (37.8)	21 (56.8)	21 (56.8)	1.000
Age	67.4 ± 12.8	67.3 ± 12.8	67.5 ± 13.0	0.808
Ever‐smoker	28 (56.7)	21 (56.8)	17 (45.9)	0.486
Comorbidities				
Hypertension	27 (36.4)	14 (37.8)	13 (35.1)	1.000
Diabetes	12 (16.2)	8 (21.6)	4 (10.8)	0.345
Old CVA	2 (2.7)	1 (2.7)	1 (2.7)	1.000
Old Tbc	10 (13.5)	7 (18.9)	3 (8.1)	0.308
COPD	6 (8.1)	3 (8.1)	3 (8.1)	1.000
Asthma	4 (5.4)	2 (5.4)	2 (5.4)	1.000
Other malignancies	16 (21.6)	9 (24.3)	7 (18.9)	0.778
Tumor markers				
CEA	26.4 ± 83.8	35.7 ± 95.7	19.2 ± 73.9	0.447
Cyfra 21–1	3.0 ± 1.9	2.9 ± 1.4	3.1 ± 2.2	0.856
Procedure duration (minutes)	42.9 ± 16.4	38.1 ± 16.2	47.6 ± 15.3	0.743
Location of PPL(s)				1.000
RUL	16 (21.6)	8 (21.6)	8 (21.6)	
RML	6 (8.1)	3 (8.1)	3 (8.1)	
RLL	20 (27.0)	10 (27.0)	10 (27.0)	
LUL	22 (29.7)	11 (29.7)	11 (29.7)	
LLL	10 (13.5)	5 (13.5)	5 (13.5)	
Nodule type				0.193
Solid	53 (71.6)	26 (70.3)	27 (73.0)	
Subsolid	15 (20.2)	6 (16.2)	9 (24.3)	
GGO	6 (8.1)	5 (13.5)	1 (2.7)	
Air‐bronchus sign	32 (43.2)	18 (48.6)	14 (37.8)	0.241
Size, mean (mm)	28.7 ± 13.2	34.5 ± 15.5	23.6 ± 7.6	< 0.001*
< 20	20 (27.0)	7 (18.9)	13 (35.1)	
20–30	28 (37.8)	11 (29.7)	17 (45.9)	
> 30	26 (35.1)	19 (51.4)	7 (18.9)	
Pleural distance (mm)	12.5 ± 12.7	10.3 ± 10.5	14.8 ± 14.4	0.102

* Significant differences (*P* < 0.05). CEA, carcinoembryonic antigen; CI, confidence interval; COPD, chronic obstructive pulmonary disease; CVA, cerebrovascular accident; GGO, ground‐glass opacity; LLL, left lower lobe; LUL, left upper lobe; RLL, right lower lobe; RML, right middle lobe; RUL, right upper lobe; TBB, transbronchial biopsy; Tbc, tuberculosis.

### Radiographic characteristics of pulmonary lesions

Radiographic characteristics of the target lesions are presented in Table 1. Most lesions were located in the left upper lobe (22, 29.7%) and were solid in appearance (53, 71.6%). The air‐bronchus sign was seen in 32 (43.2%) cases, with no significant difference between the two groups (*P* = 0.241). The mean distance from the pleura to the lesion also showed no significant difference between two groups (14.8 ± 14.4 mm vs. 10.3 ± 10.5 mm, *P* = 0.102). The mean size of the pulmonary lesion was 28.7 ± 13.2 mm, which was smaller in the R‐EBUS‐TBB group than in the TBB group (23.6 ± 7.6 vs. 34.5 ± 15.5 mm, *P* < 0.001).

### Factors associated with diagnostic yield

**Figure 2:**
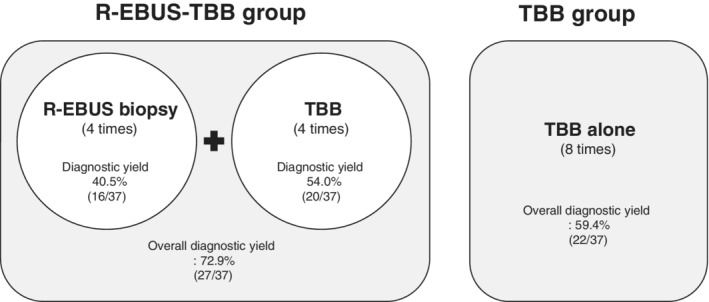
Schematic figure of diagnostic yield in two groups. * R‐EBUS, radial probe endobronchial ultrasound; TBB, transbronchial biopsy.

In the R‐EBUS‐TBB group, diagnostic yield of R‐EBUS and TBB were 40.5% and 54%, respectively. When we merged both biopsy results, overall 27 patients (72.9%) were diagnosed with lung cancer. This result was much higher than the diagnostic yield of the TBB‐only group (59.4%). The diagnostic rate of TBB was slightly higher than that of the R‐EBUS‐TBB group (Fig. 2). Among the variables studied, lung lesions with the air‐bronchus sign were associated with a higher diagnostic yield (odds ratio = 3.52 (1.17–10.62); *P* = 0.025) (Table 2).

**Table 2:** Univariate and multivariate analysis for predictors of diagnostic yield

	Univariate analysis		Multivariate analysis	
Variable	OR (95% CI)	*P*‐value	OR (95% CI)	*P*‐value
Solid nodule	0.49 (0.15–1.56)	0.223		
Nodule size ≥30 mm	0.72 (0.27–1.95)	0.526		
Air‐bronchus sign	3.30 (1.21–9.00)	0.017	3.52 (1.17–10.62)	0.025*
Cavitation	0.82 (0.21–3.24)	0.783		
Pleural distance >20 mm	0.65 (0.18–2.34)	1.000		
R‐EBUS arm	1.27 (0.48–3.33)	0.624		

* Significant differences (*P* < 0.05). CI, confidence interval; OR, odds ratio; R‐EBUS, radial endobronchial ultrasound.

### Bronchoscopic diagnosis

The bronchoscopic diagnosis of study subjects is presented in Table 3. Of the 74 patients, 49 (66.2%) were diagnosed with malignancy. Among the diagnosed cases, adenocarcinoma (49, 58.1%) was the most common. Benign conditions such as nonspecific inflammation, granuloma, and pneumonia were diagnosed in six patients (8.1%). We were unable to obtain meaningful results from 11 (29.7%) cases in the TBB group and eight (21.6%) cases in the R‐EBUS‐TBB group because the biopsy forceps were unable to approach the PPL, or insufficient tissue sample collection.

**Table 3:** Bronchoscopic diagnosis

Diagnosis	Total, No. (%)	Standard TBB, No. (%)	R‐EBUS‐TBB, No. (%)
Diagnostic	49 (66.2)	22 (59.4)	27 (72.9)
Adenocarcinoma	43 (58.1)	19 (51.3)	24 (64.8)
Squamous cell cancer	2 (2.7)	1 (2.7)	1 (2.7)
Small cell cancer	1 (1.3)	0 (0.0)	1 (2.7)
Large cell cancer	0 (0.0)	0 (0.0)	0 (0.0)
Metastasis	3 (4.0)	2 (5.4)	1 (2.7)
Nondiagnostic	25 (33.7)	15 (40.5)	10 (37.0)
Biopsy failure	19 (25.6)	11 (29.7)	8 (21.6)
Inflammation	4 (5.4)	2 (5.4)	2 (5.4)
Granuloma	1 (1.3)	1 (2.7)	0 (0.0)
Pneumonia	1 (1.3)	1 (2.7)	0 (0.0)

R‐EBUS, radial endobronchial ultrasound; TBB, transbronchial biopsy.

### Complications after the procedure

Overall, complications were encountered in seven patients (9.4%). Desaturation (6, 8.1%) was the most common complication, following by chest pain (5, 6.7%), pneumothorax (3, 4.0%), and hemoptysis (3, 4.0%). There were no life‐threatening complications such as respiratory failure and no in‐hospital mortality in the study subjects. There was no significant difference in the complications between the TBB and R‐EBUS‐TBB groups (*P* = 0.317) (Table 4).

**Table 4:** Complications of each procedure

Complications	Total, No. (%)	Standard TBB, No. (%)	R‐EBUS‐TBB, No. (%)	*P*‐value
Hemoptysis	2 (2.7)	1 (2.7)	1 (2.7)	0.667
Pneumothorax	3 (4.0)	1 (2.7)	2 (5.4)	0.750
Fever	3 (4.0)	1 (2.7)	2 (5.4)	0.600
Desaturation (SpO2 < 95%)	6 (8.1)	3 (8.1)	3 (8.1)	0.315
Chest pain	5 (6.7)	3 (8.1)	2 (5.4)	0.500
Respiratory failure	0 (0.0)	0 (0.0)	0 (0.0)	‐
Death	0 (0.0)	0 (0.0)	0 (0.0)	‐
Total cases	7 (9.4)	4 (10.8)	3 (8.1)	0.317

* Significant differences (*P* < 0.05). R‐EBUS, radial endobronchial ultrasound; SpO2, peripheral oxygen saturation; TBB, transbronchial biopsy.

## Discussion

The present study revealed that combining R‐EBUS with conventional TBB resulted in a higher diagnostic yield than TBB alone. Even though the lesion size was smaller in the R‐EBUS‐TBB group, the diagnostic yield was higher. Ultrasonographic guidance allowed the confirmation of the exact location of the lesion, thereby enabling the precise performance of biopsy. Furthermore, the acquisition of larger specimens was possible when TBB was combined with R‐EBUS.

There are several benefits and disadvantages of TBB under fluoroscopic guidance. Real‐time imaging with fluoroscopy enables visual feedback to the operator, allowing precise guidance during biopsy. However, some studies have reported that fluoroscopy does not improve specimen acquisition because it only provides two‐dimensional images. In addition, there are concerns regarding radiation exposure to the operator.^18^, ^19^

Due to the limitations of conventional bronchoscopy, efficacy of newly developed techniques of bronchoscopic biopsy, including R‐EBUS, electromagnetic navigation bronchoscopy, and thin bronchoscopy, has been reported. The overall diagnostic yield is reported to be 53.0%–82.4% using R‐EBUS. Some studies have reported increased diagnostic rates of up to 84.4% when ENB and R‐EBUS were combined.^18^, ^20^ A combination of different bronchoscopic modalities is expected to increase the diagnostic yield. However, few studies have evaluated an increase in the diagnostic yield with R‐EBUS combined with standard TBB.

Recently, a multicenter prospective study compared the diagnostic yield of standard TBB with that of R‐EBUS‐TBB^21^ and reported poor diagnostic yield despite combining R‐EBUS with standard TBB. This result may be explained by a high proportion of benign diseases among this study cohort. According to a recent study by Kim *et al*. there was no significant difference between combined use of R‐EBUS and TBB and TBB alone in the diagnosis of benign lung disease.^22^ It is difficult to determine the precise location of benign inflammatory lesions compared with that of cancer lesions usually accompanied by solid or subsolid nodules, which are typically clearly visualized on EBUS images. Thus, the presence of benign lung diseases among a study cohort may mask the benefits of R‐EBUS in identifying lung cancer. In the present study, we only enrolled patients with a high level of suspicion for lung cancer in CT images. Therefore, the proportion of patients with benign diseases was lower than that in the previous study, which may have contributed to a higher diagnostic yield with the concomitant use of R‐EBUS.

The fellowship trainees who performed the procedure in our study had a minimum experience of 5–20 months in the pulmonology department and had performed at least 100 fluoroscopy‐guided TBBs. Although the bronchoscopy skills of these operators were adequate to perform fluoroscopy‐guided TBB, they were less experienced with R‐EBUS.^23^ Because an understanding of the detailed three‐dimensional anatomical structure is essential, pathologic biopsy with R‐EBUS is a procedure that is challenging to master. A previous study had reported that over three years of experience or performance of 400 procedures is required to be able to achieve consistent success with R‐EBUS‐guided TBB.^24^ This means that even if the procedures are conducted under the instruction of a supervisor, the fellowship trainees may experience considerable difficulty in the process. However, our results showed a marked increase in the diagnostic yield with the combined use of R‐EBUS and fluoroscopic biopsy even though the examiners had not fully mastered their skills. This relative success may be attributed to the availability of guidance using fluoroscopy, which helped minimize mislocation of the radial probe by allowing visualization of the target anatomic location. Hence, in a training institution like our hospital, we expect that the combined use of the two modalities will have educational benefits as well.

The overall complication rate in the present study was similar to the previously reported complication rate of TBB^25^ and slightly higher than that reported for R‐EBUS.^26^ Although R‐EBUS was newly introduced in our department, the incidence of complications was not higher than that with TBB alone. We first identified the approximate location of the lesion under fluoroscopy, followed by precise positioning with the radial ultrasound probe, which resulted in fewer complications. Clear visualization of the bronchial and vascular structures was possible using R‐EBUS, thereby reducing the incidence of complications such as bleeding. Furthermore, we found that the air‐bronchus sign associated with PPLs was a significant predictive factor of a higher diagnostic yield, as reported in a previous meta‐analysis on R‐EBUS.^27^ If the lesion is located adjacent to the bronchus, it may be more precisely confirmed by ultrasound and contribute to a higher diagnostic yield. This combined use of fluoroscopy and the radial ultrasound probe may have contributed to the lower complication rate and higher diagnostic yield observed in the present study. Although some studies have revealed higher complication rates during the first trimester of the introduction of new techniques, our complication rate was not higher than that reported in previous studies.^28^

The overall procedure time in both groups was longer than that reported in a previous study.^29^ This was because most patients underwent EBUS‐guided transbronchial needle aspiration if mediastinal lymph nodes were noted, adding to the procedural time. However, it is noteworthy that the difference in the procedural time was not statistically significant between the groups. If the operator is skillful in TBB, a combined technique, including R‐EBUS, does not significantly prolong the duration of the procedure. This may also be a reason for the similar complication rates observed between the two groups in this study. Our findings are similar to those of a previous study by Curull *et al*. which reported that R‐EBUS under fluoroscopy was safe and did not increase the total duration of the procedure.^30^

To the best of our knowledge, the present study is the first to evaluate the impact of the introduction of R‐EBUS on the diagnostic yield in a hospital where TBB has already been implemented. We believe that this is an important finding as it offers an alternative technique that can overcome the limitations of TBB, which has a low diagnostic yield. Operators who were unfamiliar with R‐EBUS could also improve the diagnostic yield when they used it in combination with TBB.

The retrospective study design and small sample size constitute the limitations of our study. Furthermore, the lack of objective assessment data regarding the bronchoscopy skills of individual operators may be a limitation. Therefore, future prospective studies with larger sample sizes are required to determine the impact of operator skills on the diagnostic yield of the R‐EBUS‐TBB combination technique.

In conclusion, the combination of R‐EBUS and TBB resulted in a higher diagnostic yield than R‐EBUS or TBB alone. In particular, a tissue diagnosis was more likely in pulmonary lesions with the air‐bronchus sign. In institutions where fluoroscopic TBB has already been implemented, the introduction of the combination technique with R‐EBUS may increase the diagnostic yield of PPLs.

## Disclosure

The authors have no potential conflicts of interest to disclose.
